# Apoferritin and Apoferritin-Capped Metal Nanoparticles Inhibit Arginine Kinase of *Trypanosoma* *brucei*

**DOI:** 10.3390/molecules25153432

**Published:** 2020-07-28

**Authors:** Oluyomi Stephen Adeyemi, Afolake T. Arowolo, Helal F. Hetta, Salim Al-Rejaie, Damilare Rotimi, Gaber El-Saber Batiha

**Affiliations:** 1 Laboratory of Theoretical and Computational Biophysics, Ton Duc Thang University, Ho Chi Minh City 758307, Vietnam; 2 Faculty of Applied Sciences, Ton Duc Thang University, Ho Chi Minh City 758307, Vietnam; 3 Nanomedicine & Toxicology Laboratory, Medicinal Biochemistry, Department of Biochemistry, Landmark University, PMB 1001, Omu-Aran 251101, Nigeria; rotimi.damilare@lmu.edu.ng; 4 Hair and Skin Research Laboratory, Division of Dermatology, Department of Medicine, Faculty of Health Sciences, University of Cape Town, Cape Town 7925, South Africa; afolakearowolo@gmail.com; 5 Department of Medical Microbiology and Immunology, Faculty of Medicine, Assiut University, Assiut 71515, Egypt; helal.hetta@uc.edu; 6 Department of Internal Medicine, University of Cincinnati College of Medicine, Cincinnati, OH 45267-0595, USA; 7 Director for KSU Human Resources, Department of Pharmacology & Toxicology, College of Pharmacy, King Saud University, Riyadh 11451, Saudi Arabia; rejaie@ksu.edu.sa; 8 Department of Pharmacology and Therapeutics, Faculty of Veterinary Medicine, Damanhour University, Damanhour 22511, AlBeheira, Egypt; gaberbatiha@gmail.com

**Keywords:** drug discovery, medicinal biochemistry, nanomedicine, selective inhibitors, trypanosomiasis

## Abstract

The aim of this study was to explore the inhibitory potential of apoferritin or apoferritin-capped metal nanoparticles (silver, gold and platinum) against *Trypanosoma* *brucei* arginine kinase. The arginine kinase activity was determined in the presence and absence of apoferritin or apoferritin-capped metal nanoparticles. In addition, kinetic parameters and relative inhibition of enzyme activity were estimated. Apoferritin or apoferritin-capped metal nanoparticles’ interaction with arginine kinase of *T. brucei* led to a >70% reduction in the enzyme activity. Further analysis to determine kinetic parameters suggests a mixed inhibition by apoferritin or apoferritin-nanoparticles, with a decrease in V_max_. Furthermore, the K_m_ of the enzyme increased for both ATP and L-arginine substrates. Meantime, the inhibition constant (K_i_) values for the apoferritin and apoferritin-nanoparticle interaction were in the submicromolar concentration ranging between 0.062 to 0.168 nM and 0.001 to 0.057 nM, respectively, for both substrates (i.e., L-arginine and ATP). Further kinetic analyses are warranted to aid the development of these nanoparticles as selective therapeutics. Also, more studies are required to elucidate the binding properties of these nanoparticles to arginine kinase of *T. brucei*.

## 1. Introduction

Trypanosomiasis is an economically important infection in humans and animals globally. Currently, the human African trypanosomiasis also referred as sleeping sickness is endemic in 36 sub-Saharan Africa nations where more than 65 million people are at risk of the infection [1]. The African trypanosomiasis is caused by *T. brucei* in Africa, while *T. cruzi* results in Chagas disease in America [2,3,4]. On the other hand, a different type of trypanosomiasis (the African *T. brucei brucei*) affects animals and it is referred to as *Nagana* [1]. Currently, there is no effective therapy for trypanosomiasis [5,6,7]. Also, efforts toward the development of vaccine largely remain unsuccessful. The current treatments have several challenges, among which are toxic side effects, and poor effectiveness. *Trypanosoma* infection could lead to fatal outcomes if left untreated [8]. Therefore, there is a need for novel treatment strategies for trypanosomiasis. Arginine kinase could be a potential drug target not only in trypanosomes but also in the tsetse fly (the vector of disease infection and transmission) [9]. Arginine kinase is a phosphotransferase that reversibly catalyzes the formation of the phosphagen-phosphoarginine, from L-arginine and ATP as substrates [10]. Phosphoarginine supports the burst of cellular activity until metabolic events like glycogenolysis, glycolysis and oxidative phosphorylation are activated [11,12,13].

The phosphagens may act as a reservoir, not only of ATP but also of inorganic phosphate (pi) that is mostly returned to the medium by metabolic consumption of ATP [14,15]. Since there is no expression of arginine kinase in humans [16], arginine kinase serves as an attractive target for developed trypanocides. In addition, studies have shown that inhibition of arginine kinase was lethal to the parasite growth and survival [15,16]. These findings support the importance of this enzyme to the parasite survival in host cells. To this end, selective inhibitors of arginine kinase are welcome and may become candidates for the early development of trypanocides.

Previously, we reported that polyphenol-capped nanoparticles and gallotannin strongly inhibited a recombinant form of arginine kinase [17,18,19]. Specifically, nanoparticles are of interest because of their small sizes as well as the large surface to area ratio. More so, we recently showed that inorganic nanoparticles restricted the in vitro growth of various *Trypanosoma* species [20]. In addition, nanomaterials have continued to receive increasing consideration for diverse applications cutting across biology, biomedicines and the environmental science, among others [21]. In particular, gold nanoparticles in parasitology research are being investigated for detection techniques and drug development against parasitic infections. Apoferritin is a protein that usually functions to bind and store iron. It does this by combining with a ferric hydroxide-phosphate compound to form ferritin. Apoferritin from horse spleen has a hollow cage-like structure, and this has been explored as a template for the synthesis of inorganic nanoparticles including silver, gold and platinum, among others [22]. Ferritin-nanoparticles also hold prospects for biomedical applications [23]. Therefore, in the present study, we explored apoferritin-capped nanoparticles for inhibitory potential against a recombinant arginine kinase of *T. brucei*.

## 2. Results

### 2.1. Apoferritin and Apoferritin-Capped Nanoparticles Reduced the Activity of Arginine Kinase of T. brucei and Modulated Kinetic Parameters

**Figure 1:**
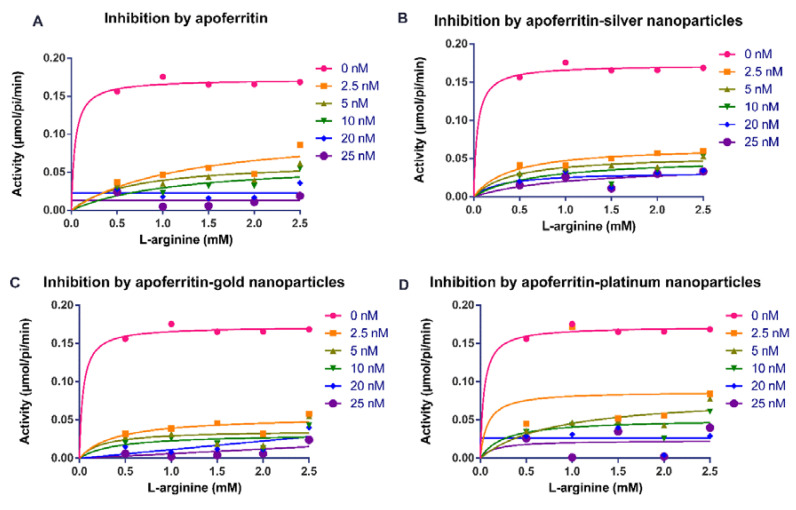
Michaelis–Menten plots for the activity (µmol/pi/min) of *Trypanosoma brucei* arginine kinase when concentration of L-arginine was varied and following incubation with (**A**) apoferritin; (**B**) apoferritin-silver nanoparticles; (**C**) apoferritin-gold nanoparticles; (**D**) apoferritin-platinum nanoparticles. Data are an average of three replicates plus/minus the corresponding error of mean (SEM).

**Figure 2:**
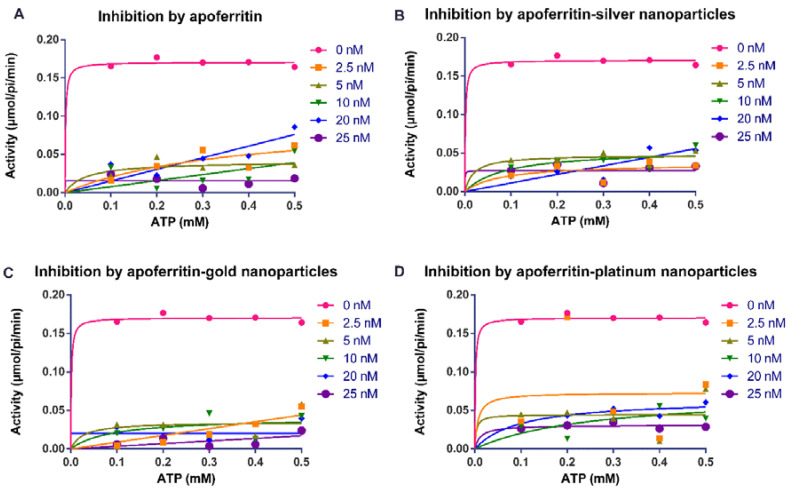
Michaelis–Menten plots for the activity (µmol/pi/min) of *Trypanosoma brucei* arginine kinase when concentration of ATP was varied and following incubation with (**A**) apoferritin; (**B**) apoferritin-silver nanoparticles; (**C**) apoferritin-gold nanoparticles; (**D**) apoferritin-platinum nanoparticles. Data are an average of three replicates plus/minus the corresponding error of mean (SEM).

Our findings showed that apoferritin and apoferritin-nanoparticles caused an appreciable decrease in the activity of arginine kinase of *T. brucei* compared with the control (Figure 1 and Figure 2). The determination of the inhibition kinetic parameters following incubation of arginine kinase with the apoferritin or apoferritin-nanoparticles was performed under two conditions. In the first condition, the L-arginine was used as a variable substrate at concentrations of 0.5–2.5 mM, and 0.5 mM ATP (Figure 1). In the second condition, the ATP concentration was varied (0.1–0.5 mM), with a fixed amount of the L-arginine at 2 mM (Figure 2). The apoferritin or apoferritin-nanoparticles concentrations used in the assay were between 2.5 and 25 nM. Findings revealed that in the first condition in which L-arginine was varied at a fixed ATP concentration (0.5 mM), V_max_ and K_m,_ respectively, were 0.169 μmol/min/mL and 0.021 mM. On the other hand, when ATP was varied, but at fixed L-arginine concentration (2 mM), V_max_ and K_m_ were 0.170 μmol/min/mL and 0.005 mM, respectively (Table 1). Nonlinear regression analysis to determine kinetic parameters and mode of inhibition in the presence of apoferritin and apoferritin-nanoparticles revealed that V_max_ decreased while, the K_m_ of the enzyme increased only for the substrate L-arginine (Table 1). Similarly, K_m_ of the enzyme for the substrate ATP increased except for apoferritin-PtNPs wherein a decrease was recorded compared with the control. Together, the data suggest an uncompetitive inhibitory interaction by the apoferritin and apoferritin-nanoparticles. In addition, inhibition constants (K_i_) values for these inhibitory interactions were in the submicromolar concentration (<0.3 nM) (Table 1), suggesting a robust binding affinity by the apoferritin and apoferritin-nanoparticles for arginine kinase of *T. brucei*. When ATP was the variable substrate, the respective K_i_ values for the apoferritin-nanoparticles were 0.057 (apoferritin-silver), 0.003 (apoferritin-gold) and 0.002 nM (apoferritin-platinum) nanoparticles. However, when L-arginine was the variable substrate, the respectively K_i_ values for the apoferritin-nanoparticles were 0.168 (apoferritin-silver), 0.160 (apoferritin-gold) and 0.245 nM (apoferritin-platinum). At the same time, apoferritin had K_i_ values of 0.001 and 0.062 nM, respectively, for ATP and L-arginine as variable substrates (Table 1).

### 2.2. Apoferritin and Apoferritin-Capped Nanoparticles Caused Significant Decreases in the Relative Activity of Arginine Kinase of T. brucei

**Figure 3:**
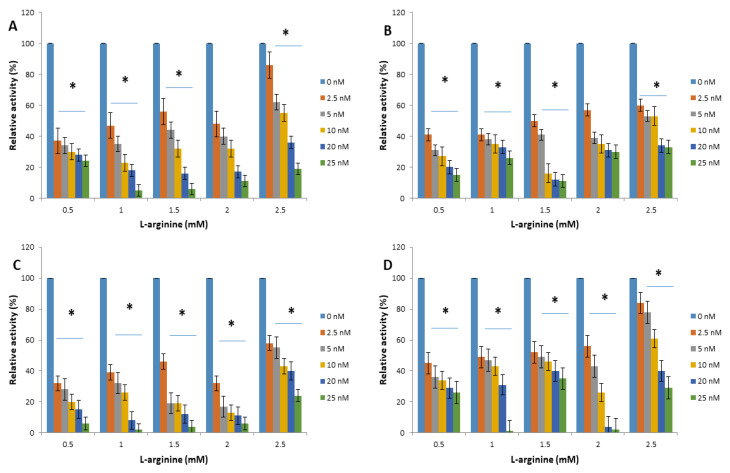
*Trypanosoma brucei* arginine kinase relative activity when concentration of L-arginine was varied and following incubation with (**A**) apoferritin; (**B**) apoferritin-silver nanoparticles; (**C**) apoferritin-gold nanoparticles; (**D**) apoferritin-platinum nanoparticles. Data are an average of three replicates plus/minus the corresponding error of mean (SEM) while at *p* < 0.05 * is significant versus no inhibitor (0 nM).

**Figure 4:**
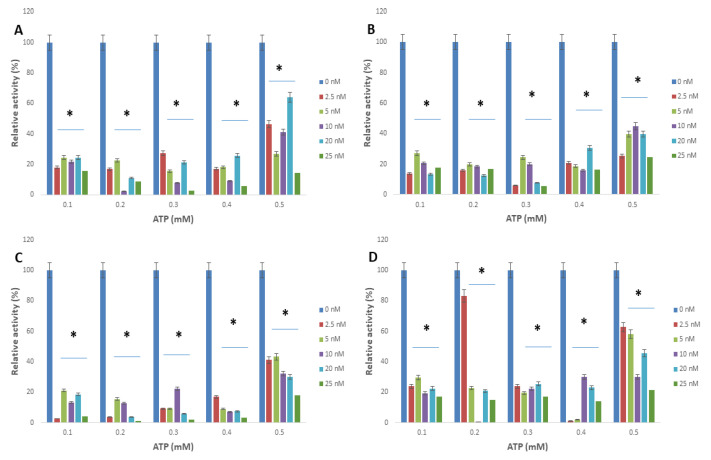
*Trypanosoma brucei* arginine kinase relative activity when the concentration of L-arginine was varied and following incubation with (**A**) apoferritin; (**B**) apoferritin-silver nanoparticles; (**C**) apoferritin-gold nanoparticles; (**D**) apoferritin-platinum nanoparticles at varied concentrations of ATP. Data are an average of three replicates plus/minus the corresponding error of mean (SEM) while at *p* < 0.05 * is significant versus no inhibitor (0 nM).

The interactions of arginine kinase with either apoferritin or apoferritin-nanoparticles led to a marked reduction (>70%) in the average relative enzyme activity compared with the control (Figure 3 and Figure 4). When L-arginine was the variable substrate, the average relative activity of the enzyme was <35% compared with the control. However, when ATP was the variable substrate, the average relative activity of the enzyme was <25% compared with the control. Overall, the data showed that the relative activity of arginine kinase of *T. brucei* was reduced by an average of >93%, >75%, >90% and >73% for apoferritin, apoferritin-silver, apoferritin-gold and apoferritin-platinum nanoparticles, respectively.

## 3. Discussion

Several investigations have shown that the arginine kinase of *T. brucei* could be a target for the development of newer trypanocides [9,10,15,16,17,18,19,24,25]. The arginine kinase of *T. brucei* is an attractive drug target simply because it is not only critical to the survival of bloodstream forms of the parasite particularly under stressful conditions, but the enzyme is completely absent in human hosts [16]. Therefore, the selective inhibition of arginine kinase of *T. brucei* may represent a viable therapeutic option.

In the present study, we explored the interactions between apoferritin-nanoparticles (Ag, Au and Pt) or apoferritin and a recombinant arginine kinase of *T. brucei*. Our findings indicate that the apoferritin-nanoparticles (Ag, Au and Pt) or apoferritin interacted with arginine kinase of *T. brucei* in a way that appreciably decreased the enzyme activity. Apoferritin reduced the activity of the arginine kinase of *T. brucei* by >90% while the apoferritin-nanoparticles decreased the relative activity of the enzyme by >70%, confirming earlier reports [17,18,19]. Due to the small sizes of these particles, both apoferritin and apoferritin-nanoparticles may offer opportunities for the selective inhibition of arginine kinase of *T. brucei*. Kinetic analysis revealed that the interactions between the Ag, Au and Pt apoferritin-nanoparticles or apoferritin were characteristic of mixed inhibition with a reduced V_max_ in the presence of the inhibitors compared with control. In addition, the small inhibition constant (K_i_) values show a strong affinity of the apoferritin or apoferritin-nanoparticles for the arginine kinase of *T. brucei*. Taken together, our present findings do not only conform to earlier reports which showed that inorganic nanoparticles of gold, silver and platinum hold prospects as therapeutic inhibitors of *T. brucei* arginine kinase in the fight against trypanosomiasis [17,18,19], but that these nanoparticles inhibited *Trypanosoma* growth [20].

Although the inhibition mechanism is yet unclear, previous reports have attributed the affinity and binding of these inorganic nanoparticles, notably Ag and Au particles, to the thiol (-SH) group of cysteine residues in protein molecules as responsible for the change in conformation and eventual inhibition or activation of enzyme activity [20,22]. So, it may be that the apoferritin-nanoparticles interacted with the arginine kinase of *T. brucei* by binding a thiol group at such a position that compromises the required whole or active site structural conformation of the enzyme and thus, enzyme activity. In a study by Pereira et al. [24,25], cysteine residues are part of the signature pattern of the guanidino kinases and these signature residues are conserved in the arginine kinase of *T. brucei* amino acids.

Our findings revealed inhibitory interactions between apoferritin-nanoparticles (Ag, Au and Pt) or apoferritin and arginine kinase of *T. brucei*. Although data do not show for the first time the inhibitory potential of apoferritin on arginine kinase of *T. brucei*, nonetheless, findings support earlier reports that nanoparticles inhibit arginine kinase. Besides, the interactions were consistent with mixed inhibition. In all, our findings warrant further analysis, including thermodynamic and in silico molecular docking studies. These studies could provide a molecular basis for the inhibition of arginine kinase by apoferritin-nanoparticles or apoferritin. The insights of the binding properties are imperative to accomplish selective inhibition of the arginine kinase of *T. brucei* as well as for the eventual development of better trypanocides. In addition, it is worth noting that in the present study, the inhibitory concentrations of the nanoparticles are in the submicromolar range. This concentration is well below the in vitro cytotoxic dose range (≥50–100 µg/mL) as reported elsewhere [26,27,28,29,30]. Furthermore, we have previously reported that the oral LD_50_ for these nanoparticles (gold, platinum and silver) is ≥1000 mg/kg bw in rats [31,32,33]. Furthermore, even though nanomaterials including metal nanoparticles hold viable prospects for biomedical applications, a key challenge is the limited understanding of how the particles interact with cellular materials.

## 4. Materials and Methods

### 4.1. Reagents

In this work, *T. brucei* arginine kinase (recombinant form) and the apoferritin-nanoparticles (silver, gold and platinum) were obtained from the Nanomedicine and Biomedical Target Laboratory, Department of Biochemistry, Microbiology and Biotechnology, Rhodes University, South Africa. The biochemical characterization of arginine kinase is reported elsewhere [17,18], while the synthesis and characterization for the apoferritin-nanoparticles are described by Sennuga et al., [34,35]. All reagents were products of Sigma, (St. Louis, MO, USA) and were of analytical grade.

### 4.2. Determination of Protein Concentration and Activity of the Arginine Kinase of T. brucei

The protein concentration was estimated by the Bradford method [36], while the enzymatic activity of *T. brucei* arginine kinase was confirmed as described elsewhere [17,18,19]. For the determination of enzyme activity, the assay medium contained Tris-HCl buffer (100 mM, pH 8.6), L-arginine (2 mM), ATP disodium salt (0.5 mM), mercaptoethanol (10 mM), magnesium sulfate (200 mM) and 0.002 mmol of the enzyme. After incubation at 30 °C for 5 min, 2.5% TCA (trichloroacetic acid) was added to stop the reaction. After that, the reaction mixture was heated at 100 °C in a heating block and cooled on ice for 2 min. Inorganic phosphate (pi) determination reagent (fresh mixture of 9% ascorbic acid and acidic ammonium molybdate) was added to the reaction mixture, and the absorbance was recorded at 700 nm on a microplate reader (Synergy Mx, Biotek Instruments, Inc. Winooski, VT, USA). The enzyme was absent in the control assay samples. The concentration of pi produced was extrapolated from a calibration curve of a standard. A unit of activity of the arginine kinase equals the amount of the enzyme that catalyzes the production of 1 µmol pi per minute.

### 4.3. Assay to Determine Arginine Kinase Activity in the Presence of Apoferritin or Apoferritin-Nanoparticles

The arginine kinase activity in the presence of apoferritin or apoferritin-metal nanoparticles was determined as described above. The inhibitory effect on the enzymatic activity of arginine kinase of *T. brucei* was studied at varying concentrations (2.5 to 25 nM) of the apoferritin, apoferritin-metal capped nanoparticles (silver, gold, and platinum nanoparticles).

### 4.4. Data Presentation and Statistical Analysis

Data were analyzed using the Michaelis–Menten plots and the nonlinear regression (GraphPad Prism 6.0, San Diego, CA, USA) analysis was used to determine the kinetic parameters and the K_i_ values. Data are presented as mean of three replicates ± standard error of mean (SEM). In addition, comparisons of mean values relative to control were done using ANOVA and Dunnett’s post hoc test, while *p* < 0.05 was taken as statistically significant.

## Figures and Tables

**Table 1:** Kinetic parameters estimated for the interaction between arginine kinase of *Trypanosoma brucei* and apoferritin or apoferritin-nanoparticles.

	* Vmax (µmol/sec)	** Vmax (µmol/sec)	Km (L-arginine) (mM)	Km (ATP) (mM)	* Mean Ki (nM)	** Mean Ki (nM)
Control Assay	0.169 ± 0.004	0.170 ± 0.010	0.021 ± 0.01	0.005 ± 0.00	-	-
Apoferritin	0.012 ± 0.001	0.016 ± 0.001	0.041 ± 0.00	0.015 ± 0.00	0.062 ± 0.01	0.001 ± 0.00
Apoferritin-AgNPs	0.044 ± 0.002	0.034 ± 0.000	0.041 ± 0.01	0.011 ± 0.00	0.168 ± 0.03	0.057 ± 0.00
Apoferritin-AuNPs	0.067 ± 0.006	0.016 ± 0.001	0.044 ± 0.00	0.015 ± 0.00	0.160 ± 0.00	0.003 ± 0.00
Apoferritin-PtNPs	0.024 ± 0.007	0.030 ± 0.001	0.043 ± 0.00	0.001 ± 0.00	0.245 ± 0.03	0.002 ± 0.00

* L-arginine as a variable when ATP was fixed at 0.5 mM, while **ATP as a variable substrate while L-arginine was fixed at 2 mM.
